# Development of emulsion films based on bovine gelatin‐nano chitin‐nano ZnO for cake packaging

**DOI:** 10.1002/fsn3.1424

**Published:** 2020-01-23

**Authors:** Samar Sahraee, Jafar M. Milani, Babak Ghanbarzadeh, Hamed Hamishehkar

**Affiliations:** ^1^ Department of Food Science and Technology Sari Agricultural Sciences and Natural Resources University Sari Iran; ^2^ Food Science and Technology Faculty of Agriculture University of Tabriz Tabriz Iran; ^3^ Drug Applied Research Center Tabriz University of Medical Sciences Tabriz Iran

**Keywords:** active packaging, chitin, emulsion film, gelatin film, nanocomposite, ZnO nanoparticle

## Abstract

This research extends the effect of packaging with bovine gelatin, gelatin nanocomposite (GN), gelatin emulsion (GE), two layers gelatin nanocomposite and gelatin emulsion (GNE), and polyethylene (PE) films on sponge cake properties during storage at 25°C and 55 ± 2% RH. In this regard, water vapor permeability (WVP) and oxygen permeability (OP) of films were compared. Then, moisture content, acidity, peroxide value, texture profile, organoleptic properties, and fungal growth of packed cakes were determined. Results showed that the addition of nanoparticles could reduce the water vapor permeability from 9.680 ± 0.460 × 10^–10^ (g m/sm^‐2^Pa^‐1^) for net gelatin film to 6.067 ± 0.337 × 10^–10^ (g m/sm^−2^ Pa^−1^) for gelatin nanocomposite film and oxygen permeability from 39.262 (cm^3^μm/ m^2^dkPa) for net gelatin film to 29.645 (cm^3^μm m^−2^ dkPa) for nanocomposite film. However, GNE films had the highest barrier properties. Results of acidity and peroxide values of cakes admitted the sufficiency of GNE films for sponge cakes packaging. In addition, antifungal properties of nanoparticles led to less fungal growth on cakes packed in GNE films. The cakes packed in GNE films own more organoleptic and texture acceptability than the ones packed in other films. Generally, according to the results GNE films are acceptable for packaging of sponge cakes which contain no preservative because this packaging can prevent fungal growth for a longer time and even more can maintain the cake chemical and organoleptic quality.

## Graphical abstract

This research extends the effect of packaging with gelatin, gelatin nanocomposite, gelatin emulsion, two layers gelatin nanocomposite and gelatin emulsion, and polyethylene films on sponge cake properties during storage at 25°C and 55 ± 2% RH. Addition of nanoparticles to gelatin films has improved barrier properties of the films, but it was not sufficient. The results of moisture content, peroxide value, acidity, texture analysis, and fungal growth admitted that two layers nanocomposite emulsion films could be a substitute for polyethylene films.

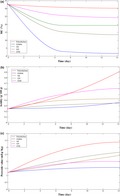

## 1 INTRODUCTION

Bakery products are the important portions of daily diet. These products including breads, cakes, biscuits, and donuts usually are comprised of complex carbohydrates, protein sources, lipids, vitamins, and minerals (Soukoulis et al., 2014). Meanwhile, cakes are of popular bakery products because of nutraceutical properties, overall perception, and low price. Although the shelf life of cakes is longer than breads, mold growth and lipid oxidation are still major problems of cake preservation (Lu, Lee, Mau, & Lin, 2010). Several methods have been used for increasing bakery products shelf life such as applying antioxidant and antimicrobial additives, and controlled or active packaging (Chakravartula, Cevoli, Balestra, Fabbri, & Rosa, 2019). Recently, biodegradable packaging has been attended because of environmentally friendly properties, produced from bio‐waste by‐products, low cost, and capability to carry active component to preserve foods (Soukoulis et al., 2014). However, a major challenge for industrial application of these polymers is their low barrier properties against water molecules (Zheng, Tajvidi, Tayeb, & Stark, 2019).

One of the most important source of biodegradable films is gelatin. This material is interested because of low cost, availability, and film forming properties but its disadvantage is high water vapor permeability (Marvizadeh, Oladzadabbasabadi, Nafchi, & Jokar, 2017).There are different ways to improve barrier properties of biodegradable films such as different film production processes, applying cross‐linking agents, plasticizers, and filling components such as nanoparticles (Araghi et al., 2015). Recently, researchers have studied effect of different nanoparticles on biodegradable films properties (Marvizadeh et al., 2017; Nafchi, Nassiri, Sheibani, Ariffin, & Karim, 2013). In this case, nano chitin (N‐chitin) is one of the compatible nanoparticles with carbohydrate and protein‐based polymers that not only can improve physical properties of films, but also can add antimicrobial properties to biopolymer (Sahraee, Milani, Ghanbarzadeh, & Hamishehkar, 2017). On the other hand, metal oxide nanoparticles such as nano ZnO (N‐ZnO), TiO_2_, MgO, and CaO are interested in their high stability to temperature of process, diffusivity from packaging to food, being safe for animals and human and antimicrobial properties (Nafchi, Moradpour, Saeidi, & Alias, 2014; Shankar, Teng, Li, & Rhim, 2015).

Therefore, substituting biodegradable packaging for bakery products instead of synthetic polymers may be a substantial action in order to decrease waste pollution in environment and fuel usage for producing olefin polymers. Even more, because of high functionality of biodegradable polymers, addition of preservatives to bakery products may reduce. The objective of the present research was to apply gelatin nanocomposite films containing N‐chitin and N‐ZnO for packaging of sponge cake and compare the shelf life and quality of cakes with the ones packed in polyethylene films.

## 2 MATERIALS AND METHODS

### 2.1 Materials

For film preparation materials, bovine gelatin was supplied by Merck Chemical Co. (Darmstadt, Germany), with bloom number of 200 and density of 1,358 kg/m^3^. N‐ZnO powder with size of 10–30 nm was purchased from Nano SANY Co. N‐chitin gel was bought from Nano‐Novin Co. with 1.5% dry matter and particle size of 50–70 nm. Liquid glycerol and 50% glutaraldehyde were provided by Sigma Chemical Co. For cake ingredients, wheat flour (extraction rate of 72%), sugar, eggs, oil, vanilla, and baking powder were provided from a local market, Tabriz, Iran.

### 2.2 Chemicals

Sodium hydroxide, phenolphthalein, n‐hexane, acetic acid, chloroform, sodium thiosulphate, and potassium iodide were purchased from Sigma Aldrich. In order to study antifungal activity, SDS Agar medium was supplied from Quelab.

### 2.3 Film preparation and characterization

#### 2.3.1 Film preparation

An aqueous solution of ZnO nanoparticles was prepared through dispersing 5% (based on dry gelatin) of N‐ZnO powder in 100 ml distilled water and was stirred on a magnet stirrer at 30°C for 1 hr. Then, the solution was sonicated using a high‐intensity ultrasonic processor (Heidolph) at periodic pulsing of 120 s on and 15 s off and at an amplitude of 80% with 0.5 cycle per second. Subsequently, 5% N‐chitin (based on dry gelatin) was added to the solution and mixed for 1 hr further, followed by sonication in an ultrasonic bath (Parsonic 30S, Pars Nahand engineering Co.) for 30 min. Gelatin (4 g/100 ml) was dissolved in this solution by mixing for 30 min at room temperature followed by stirring on a hot plate at 55°C for 30 min. Later, the solution was cooled to 35°C and 30% glycerol as plasticizer and 1% glutaraldehyde as cross‐linking agent was added and mixed for 30 min. Finally, the film solution was cast on 16 cm diameter Teflon‐coated dishes and dried for 48 hr at room temperature. Also, net gelatin films were produced through the same method without adding nanoparticles. In order to make ready emulsion films, 4 g/100 ml gelatin was swelled in water for 30 min and 30% (based on dry gelatin) corn oil and Tween‐80 (2 g/100 g oil) as emulsifier were added to the solution and heated up to 55 ± 5°C for 30 min. Then, the mixture was cooled to 35°C and plasticizer and cross‐linking agent were added as described above and mixed for 30 min further. Finally, the solution was cast on Teflon‐coated dishes and dried. Four kinds of packaging films were prepared in order to pack the cakes: net gelatin films, 5% N‐chitin/ 5% N‐ZnO/gelatin films (GN), gelatin emulsion films (GE), and 5% N‐chitin/ 5% N‐ZnO/gelatin as first layer and gelatin emulsion film as second layer (GNE), and polyethylene films as control (Sahraee, Milani, Ghanbarzadeh, & Hamishekar, 2016).

#### 2.3.2 Water vapor permeability

Water vapor permeability (WVP) of films was measured according to the method of ASTM E96/E96M‐16 (2016) with some modifications (Zahedi, Ghanbarzadeh, & Sedaghat, 2010). In this method, 3 g CaSO_4_ salt was filled in glass vials (1.5 cm diameter and 4 cm depth). This salt induced 0% RH in the vials. Then, disks of film samples were fixed with vial doors on top of them. There was a hole of 4 mm diameter in each door to let gas exchange through the films. The initial weigh of vials were measured and then put in the desiccators containing saturated K_2_SO_4_ (RH = 97%). The vials were weighed at 24 hr intervals. That way, water vapor transmission rate (WVTR) was calculated from the slope of curve of weight difference versus time. Subsequently, the WVP (g/m s^‐1^ Pa^−1^) of film samples were determined according to equation (1):

(1) WVP=(WVTR×L)/ΔP

where L is the average thickness of films, and ∆*P* is the water vapor pressure difference between two sides of films.

#### 2.3.3 Oxygen permeability of films

Oxygen permeability (OP) of films was determined according to the standard method of ASTM D3985–17 (2017) with a modular system of Ox‐Tran 2/20 Ml (Modern Controls Inc.) at 25°C and 55 ± 2% RH. The film samples were fixed on a stainless steel mask. One side of film was in exposure of nitrogen gas and the other side was in contact with oxygen gas. Both sides of films were set at the same temperature and humidity. As oxygen permeated the film, it transferred to calorimetric detector and produced electrical flow, which its intensity was depended on amount of oxygen flowing to the detector per time (Hong and Krochta, 2006).

### 2.4 Cake preparation and properties

#### 2.4.1 Cake preparation

The cake samples were prepared according to the method of Lu et al. (2010) by some modifications. The ingredients were mixed as following formula: 150 g flour, 150 g sugar, 6 eggs, 40 g oil, 1.70 g vanilla, and 0.90 g baking powder. After preparing the dough, it was transferred to a cake mold and baked for 40 min at 180°C in oven. After baking, it was covered with a sterile aluminum paper and hold in a laminar hood in order to be cooled. Then, 5 × 5 cm^2^ pieces of cakes were packaged in gelatin nanocomposite films and polyethylene films as control and stored at 55 ± 2% RH and 25°C. The shelf life investigations of packed cakes were done in 0, 7, 14, 21, and 28 days of storage.

#### 2.4.2 Moisture content of cake

Moisture content of cake samples (crumb) was determined according to AACC 44‐15A (AACC, 2000). According to this method, pieces of 2 × 2 cm^2^ of cakes weighed before and after drying at 103°C for 24 hr and the percentage of weight loss was reported as moisture content of the cake.

#### 2.4.3 Extraction of cake's lipid

Lipid extraction from cake samples was necessary to determine peroxide value and acidity of cakes’ lipid during storage. In this regard, 100 g of cake samples was immerged in 200 ml n‐hexane as a solvent and mixed thoroughly to become crashed and exposed better to the solvent. The mixture was held until the upper solvent became clear and then filtered through filter paper (Whatmann No. 1). Subsequently, the solvent was evaporated by rotary at 50°C, and the extracted lipid was taken for next experiments (Lu et al., 2010).

#### 2.4.4 Peroxide value

In order to determine the rate of lipid oxidation during the storage of cakes packed in different kinds of polymers, peroxide value (PV) was measured at 0, 7 and 14 days of storage according to the method of AOCS (AOCS Cd8b‐90, 2017). In this regard, 2 g of extracted lipid was weighed in 250 ml flask and 30 ml of acetic acid and chloroform solution (3:2 acetic acid to chloroform volume ratio) was added. Then, 0.5 ml of saturated solution of potassium iodide was incorporated in the solution and held for 1 min in a dark place. Subsequently, after addition of 30 ml distilled water to the solution, it was titrated by sodium thiosulfate (0.01 N) in the presence of starch solution as the indicator. The peroxide value was calculated according to the following equation (2):

(2) $$P=\frac{N\times V\times 100}{W}$$

where*N* is the sodium thiosulfate normality, *V* is the volume of sodium thiosulfate used for titration, and *W* is the weight of lipid.

#### 2.4.5 Free fatty acids

One of the rancidity symptoms of food's lipid is increasing its free fatty acid content. Based on this fact, the acidity of cakes lipid was measured at 0, 7, and 14 days of storage according to the method of AOCS (AOCS Ca5a‐40, 2017). According to this method, 2 g of lipid was mixed with 30 ml of neutralized ethanol and titrated with sodium hydroxide solution (0.01 N) in presence of phenolphthalein as indicator. The acidity of cake lipid was calculated by:

(3) $$\text{Acidity}=\frac{28.2\times N\times V}{W}$$

where *N* is normality of sodium hydroxide solution, *V* is the volume of titrated sodium hydroxide, and *W* is the weight of lipid.

#### 2.4.6 Texture profile analysis of cakes

In order to study the effect of packaging polymer on texture properties of cake samples after 0, 7, and 14 days of storage, texture profile analysis of cakes was performed by an Instron universal testing machine (Texture Pro CT V1.6 Build, Brookfield Engineering Labs. Inc.).Cube pieces of cakes (4 × 4 × 4 cm^3^) compressed using a cylinder probe (TA25/1,000, D = 1.245 mm) at room temperature. The samples were compressed in two cycles to 40% of their initial heights with a load of 100N and speed of 1mms^‐1^.The springiness, cohesiveness, hardness, and resilience of the cake samples were determined as the mean of triple measurements.

#### 2.4.7 Antimicrobial activity of films

In order to assess the antifungal properties of packaging films on cakes, mold and yeast counts have been done according to the methods described in AOAC, 2014. Aseptically, the sample was diluted 1:10 with dilution water and stomached for 2 min. About 1 ml of diluted sample was carefully transferred on the surface of solidified medium in plate. Then, the suspension was distributed evenly using gentle movement of pipette on the medium. The door of plate was closed and left for 1 min to allow the suspension be absorbed to the medium. The yeasts and molds were counted after 5 days of incubation in 25°C.

#### 2.4.8 Sensory evaluation

Sensory evaluation of cake samples was implemented to assess the effect of different packaging polymers on quality maintenance of cakes during 7 days of storage at ambient temperature. In this regard, 20 panelists including 7 men and 14 women were asked to test cake samples which were labeled with a three digit random numbers. Each panelist filled an evaluation form, ranking the quality attributes of appearance, color, odor, texture, overall acceptability by applying a 5‐point hedonic scale (1 = dislike extremely and 5 = like extremely). The results are the average of these ratings (Altamirano‐Fortoul, Moreno‐Terrazas, Quezada‐Gallo, & Rosell, 2012).

### 2.5 Statistical analysis

The results of all experiments were stated as mean ± standard deviation. The one‐way analysis of variance (ANOVA) was applied for analyzing the data. For post hoc comparisons, Duncan's test was considered. In order to study gelatin nanocomposite film's properties, Tukey's test was applied. SPSS 16.0 (SPSS) has been used for analyzing the results. In all experiments, the significant level was *p* < .05.

## 3 RESULTS AND DISCUSSION

### 3.1 Water vapor permeability of films

Water vapor permeability of gelatin, GN and GE films were shown in Table 1. Statistical analysis of data showed significant differences between WVP of the films. Net gelatin films had high WVP because of hydrophilic nature of the films. However, incorporation of N‐chitin and N‐ZnO increased barrier properties of films against water vapor. Since WVP of films is dependent to water molecules solubility and diffusion across the film, nanoparticles can reduce this permeability by increasing cross‐linkage between polymer chains and filling the porosity of matrix (Rouhi, Mahmud, Naderi, Ooi, & Mahmood, 2013). Also, Kanmani and Rhim (2014) reported that addition of N‐ZnO in different polymers reduced WVP by increasing overall hydrophobicity of films and inducing tortuous passway across water vapor.

**Table 1:** Water vapor permeability (WVP) and oxygen permeability (OP) of gelatin, GN, GE, and polyethylene films

Film sample	WVP (× 10^–10^ g m/sm^−2^Pa^−1^)	OP (cm^3^μm/m^2^dkPa)
Gelatin	9.680 ± 0.460^d^	39.262 ± 2.544^b^
GN	6.067 ± 0.337^b^	29.645 ± 1.980^a^
GE	7.680 ± 0.750^c^	50.473 ± 4.001^c^
Polyethylene	3.600 × 10^–3a^ (Thakur et al., 2017)	1,866.240^d^ (Thakur et al., 2017)

Note

Similar superscripts in each column show no significant difference between mean values (*p* < .05).

Addition of oil to film formulation led to less WVP than gelatin and GN films. The result may be due to the enhancing hydrophobic property of emulsion films and reducing gelatin films tendency toward water molecules (Otoni, Pontes, Medeiros, & Soares, 2014). Since gelatin has amphiphilic property, the added corn oil to the films could interact well with film chains and high concentrations of oil did not cause phase separation in polymer. Homogenous distribution of oil molecules through the film led to less WVP and inhibition of creating voids in the interface of hydrophilic‐hydrophobic surfaces. Although addition of oil decreased WVP of gelatin films, the barrier property of gelatin emulsion films was not as well as synthetic films like polyethylene with WVP of 3.6 × 10^–13^ g msm^–2^ Pa^–1^ (Thakur et al., 2017).

### 3.2 Oxygen permeability of nanocomposite emulsion films

Oxygen permeability of films (OP) was shown in Table 1. Films owning hydrophilic nature have high water vapor permeability, but their barrier property against gases like oxygen and carbon dioxide is good. Nanoparticles incorporation in gelatin films had significantly decreased oxygen permeability of them. Generally, enhancing crystal phase of film, orientation, molecular weight, and cross‐linking of chains lead to reduce oxygen permeability of films (Li, Cao, Pei, Liu, & Tang, 2019). Based on the fact that nanoparticles can increase cross‐linking and cohesiveness of polymer chains; they improve oxygen barrier property of films.

Addition of corn oil to film formulation increased OP of GE films in comparison with net gelatin films. The reason may be hydrophobic nature of lipids which increase oxygen transfer through the films. Also, Bertan, Tanada‐Palmu, Siani, and Grosso (2005) found similar results when studied gelatin and triacetin films gas permeability. They reported that formation of microholes in film matrix by incorporation of lipids in gelatin films can lead to more gas transfer across the films.

Comparison of OP of gelatin nanocomposite films with that of polyethylene films revealed that OP of biodegradable nanocomposite films is very low. The OP of polyethylene films at 25°C was about 1866.240 cm^3^μm m^–2^ dkPa (Thakur et al., 2017) whereas the GN films had the OP of 26.494 cm^3^μm m^–2^ dkPa.

### 3.3 Moisture content of cakes

**Figure 1:**
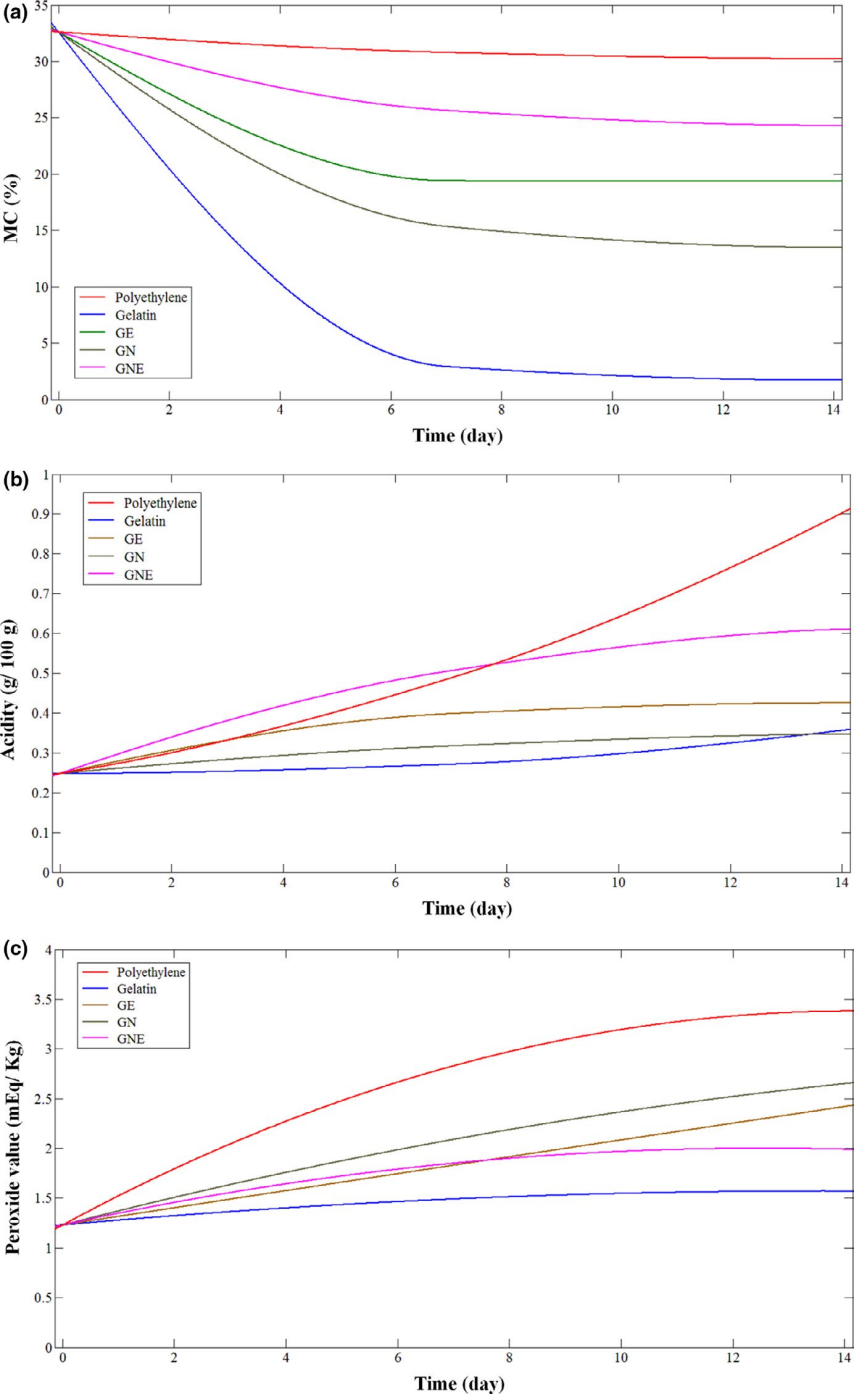
(a) Moisture content (MC) (%), (b) Acidity (g oleic acid/ 100 g extracted oil), (c) Peroxide value (mEq peroxide/ Kg extracted oil) of sponge cake packed in gelatin, GE, GN, GNE, and polyethylene films at 25°C

The moisture content (MC) of cakes packed in gelatin, GE, GN, GNE, and polyethylene films stored at 25°C and RH = 55 ± 2% for 7 and 14 days have been shown in Figure 1a. Results have shown that MC of cakes was significantly affected by packaging film. MC of cakes packed in polyethylene films did not significantly alter after 14 days of storage. Nevertheless, net gelatin films could not prevent moisture loss of cakes and their MC drop‐off from 32.60 ± 0.7% for fresh cakes to 2.91 ± 0.93% after 7 days of storage. Therefore, net gelatin films did not possess proper moisture barrier property for food packaging. Also, former studies have shown that net gelatin films did not have a high water vapor barrier property (Sahraee et al., 2017). Comparison of gelatin films containing N‐chitin and N‐ZnO with net gelatin films as a packaging film showed that GN had preserved MC of cakes better than net gelatin films. Also, Amjadi et al. (2019) found that addition of ZnO and chitosan nanoparticles to the gelatin film significantly decreased its water vapor permeability.

Recent researches have shown that N‐chitin and N‐ZnO could improve barrier properties of gelatin films against water vapor through filling property and inducing a tortuous passway across the water molecules (Shankar et al., 2015, Rhim, 2015). As it can be seen in Figure 1, applying gelatin emulsion films as a second layer for gelatin films has improved barrier property of degradable packaging and GNE had the best barrier property after polyethylene.

### 3.4 Acidity of cakes

Free fatty acid (FFA) values of packed cakes packed in different films and stored at 25°C and RH = 55 ± 2% for 7 and 14 days have been shown in Figure 1b. FFA values of cakes were measured in order to determine the rancidity of cakes under the influence of packaging condition. Generally, FFA of foods is increased by hydrolysis of triglycerides in exposure to moisture or enzymatic activity of microorganisms. Statistical analysis has declared that there is no significant difference in acid value of cakes packed in net gelatin and GN films after 7 and 14 days of storage. The result may be due to the inefficiency of these polymers to avoid moisture loss of cakes and lack of moisture prevents triglycerides hydrolysis (Soukoulis et al., 2014). However, the acidity of samples packed in GE was higher than net gelatin films which was the result of higher moisture barrier of this packaging. On the other hand, the acidity of cake samples packed in polyethylene and GNE did not change after 7 days of storage. This may be because of similar moisture content of cakes packed in these films. However, the acidity became significantly different for cakes packed in polyethylene and GNE after 14 days, which probably was due to more fungal growth in polyethylene packaging than GNE films.

### 3.5 Peroxide value of cakes

The effect of different packaging polymers on cake samples’ peroxide value (mEq peroxide/Kg extracted oil from cakes) has been shown in Figure 1c. Generally, lipid oxidation of foods is affected by UV light, temperature, moisture content, metal ions, and oxygen exposure (Wu et al., 2013).

Results have shown that there is no significant difference between peroxide value of fresh and stored cakes packed in net gelatin films for 7 and 14 days of storage. The reason maybe good barrier property of gelatin films against oxygen and UV light (Sahraee et al., 2016), and moisture loss of packed cakes in net gelatin films up to a_w_ (a_w_ = 0.2–0.4) that lipid oxidation speed would decrease to the minimum. It is obvious that the peroxide value of cakes packed in polyethylene films is higher than other samples. This would be because of low barrier property of polyethylene against UV light and oxygen gas, high moisture content of cakes, enough a_w_ for oxidation process, and fungal growth and production of lipoxygenase enzyme in cakes, which could produce hydroperoxides. Even more, peroxide value of cakes packed in GE and GN films during 14 days of storage did not changed significantly. It may be due to the similar barrier property of GE and GN films against water vapor, oxygen, and UV light. According to the results of Table 1, the WVP and OP of the GN films were less than GE films. But the condition of the WVP and OP tests are different from when the film is used for cake packing. In WVP test, films are in exposure of special water vapor pressure difference. Also in OP test, films are conditioned in 55% RH and oxygen gas pressure was imposed to the films. But during the application of films for packaging the cakes, the situation is different. Different water vapor and oxygen pressure was in either sides of the films, and also in some parts, films were in exposure of cakes and some components of the cake like its fat was transferred on the film. All this differences caused the peroxide value of the cakes packed in these films changed in similar way. Therefore, because the causes of peroxide value's change in cakes were oxygen, water vapor, and UV light, similar peroxide values led to concluding of similar water vapor, oxygen and UV light exposure of cakes.

Finally, the results have declared that also GNE films can preserve the moisture content of cakes better than other polymers, but because of good barrier property against oxygen and antifungal properties of N‐chitin and N‐ZnO in gelatin nanocomposite films, it can maintain the peroxide value of cakes at lower levels than other compared polymers. These findings are in accordance with Panea, Ripoll, González, Fernández‐Cuello, and Albertí (2014) studies which stated that incorporation of N‐ZnO and N‐Ag in LDPE decreased breast lipid oxidation. Even more, Ejaz, Arfat, Mulla, and Ahmed (2018) applied gelatin film containing N‐ZnO and clove essential oil for packaging shrimps. They found that existence of nanoparticles decreased the oxygen permeability of films and inhibited the increase of peroxide value of shrimps.

### 3.6 Antifungal properties of films

Antifungal properties of the films was investigated by comparing fungal growth on cake samples packed in gelatin, GE, GN, GNE, and polyethylene films (Table 2). Since the cakes that are packed in net gelatin films have dried after 3 days of storage and their moisture content has reached to 2.91 ± 0.92% and 1.70 ± 0.54% after 7 and 14 days, no fungal growth has occurred in 7, 14, 21, and 28 days. Even more, incorporation of nanoparticles to gelatin films has decreased water vapor permeability, but the moisture content of the cakes packed in GN films has decreased to 13.48 ± 0.45% after 14 days. Therefore, the water activity of cakes was less than the minimum a_w_ for fungal growth on cakes. The comparison of fungal growth on cakes packed in polyethylene, GE, and GNE films has shown that fungal growth on cakes packed in polyethylene was more than cakes in GE and GNE after 7, 14, 21, and 28 days. The reason for less microbial growth on cakes packed in GE films may be less moisture content of cakes and less oxygen permeability of GE films.

**Table 2:** Yeast and mold count of sponge cake packed in gelatin, GE, GN, GNE, and polyethylene films at 25°C

Packaging film	CFU/g of sponge cake				
	Storage time (days)				
	0	7	14	21	28
Gelatin	0^aA^	0^aA^	0^aA^	0^aA^	0^aA^
GE	0^aA^	3.33 ± 5.77^bB^	16.67 ± 5.77^cC^	20.00 ± 10.00^cD^	UC^dE^
GN	0^aA^	0^aA^	0^aA^	0^aA^	0^aA^
GNE	0^aA^	0^aA^	3.33 ± 5.77^bB^	10.00 ± 10.00^bC^	26.66 ± 11.54^cD^
Polyethylene	0^aA^	13.33 ± 5.78^cB^	UC^dC^	UC^dC^	UC^dC^

Note

The results are mean ± *SD* values of three replicates. The same lower case superscripts in each column and uppercase superscript in each row show no significant difference between values by Tukey's test at 5% probability.

However, results obviously showed that GNE films not only preserves the moisture content of cakes but also because of including N‐chitin and N‐ZnO is a functional nanocomposite film with antifungal properties that can increase cake shelf life by reducing microbial growth. In this regard, applying two layers packaging can accomplish the property of water vapor barrier by gelatin emulsion films and support antifungal and better physicochemical properties by gelatin nanocomposite films (Noshirvani, Ghanbarzadeh, RezaeiMokarram, & Hashemi, 2017; Sahraee et al., 2017).

### 3.7 Texture profile analysis of cakes

Effect of packaging film and storage time on texture properties of cake samples stored at 25°C for 0, 7, and 14 days was determined. In this regard, it should be mentioned that cakes packed in gelatin films became dried after 3 days of storage and their texture was very hard for texture analyzing. The effect of storage time on cake hardness packed in polyethylene films was descending, but for other films was ascending (Table 3). The reason may be due to less moisture loss of cakes in polyethylene packaging and softening of the cake texture by moisture condensation. However, increasing cake hardness in other packaging films may be associated to staling of cake and loss of moisture during time (Khoshakhlagh, Hamdami, Shahedi, & Le‐Bail, 2014). Even more, the comparison of the effect of different packaging films on cakes hardness showed that after 7 and 14 days of storage the hardness of cakes in GNE was significantly lower than others in degradable packaging. The reason may be better moisture preservation and less oxygen permeability of this film. The highest hardness belonged to cake packed in GN films, may be because of more water vapor permeability through the films.

**Table 3:** Texture profile analysis of sponge cake packed in gelatin, GE, GN, GNE, and polyethylene films at 25°C

Film	Storage time (days)	GE	GN	GNE	Polyethylene
Hardness (g)	0	2,772.00 ± 246.05^aA^	2,772.00 ± 246.05^aA^	2,772.00 ± 246.05^aA^	2,772.00 ± 246.05^aC^
	7	3,298.00 ± 166.25^bB^	4,512.00 ± 1,120.6^cB^	3,146.00 ± 319.58^bB^	2,248.00 ± 761.52^aB^
	14	8,028.00 ± 2,089.53^cC^	9,132.00 ± 1793.01^cC^	5,248.00 ± 974.91^bC^	1,623.00 ± 327.65^aA^
Springiness (mm)	0	13.61 ± 0.24^aBC^	13.61 ± 0.24^aB^	13.61 ± 0.24^aC^	13.61 ± 0.24^aA^
	7	12.40 ± 0.30^aAC^	12.74 ± 0.61^aA^	12.82 ± 0.39^aB^	13.4 ± 0.37^aA^
	14	14.30 ± 3.32^aC^	12.30 ± 2.12^aB^	12.67 ± 0.48^aA^	12.08 ± 0.57^aB^
Cohesiveness	0	0.42 ± 0.04^aA^	0.42 ± 0.04^aA^	0.42 ± 0.04^aA^	0.42 ± 0.04^aA^
	7	0.49 ± 0.01^bB^	0.52 ± 0.02^cB^	0.48 ± 0.02^abB^	0.44 ± 0^aB^
	14	0.59 ± 0.02^bC^	0.62 ± 0^cC^	0.58 ± 0.02^bC^	0.54 ± 0.03^aC^
Resilience	0	0.26 ± 0.02^aC^	0.26 ± 0.02^aC^	0.26 ± 0.02^aC^	0.26 ± 0.02^aB^
	7	0.22 ± 0.02^aB^	0.23 ± 0.01^aB^	0.25 ± 0.02^aB^	0.28 ± 0.01^bC^
	14	0.16 ± 0.02^aA^	0.17 ± 0.01^abA^	0.20 ± 0.02^cA^	0.19 ± 0.01^bcA^

Note

The results are mean ± *SD* values of three replicates. The same lower case superscripts in each column and uppercase superscript in each row show no significant difference between values by Tukey's test at 5% probability.

The results of cohesiveness were in accordance with hardness (Table 3). In this case, the cakes packed in polyethylene and GNE films were less cohesive than the ones in GE and GN films. Also, the effect of storage time on cohesiveness of cakes packed in different films was ascending. However, different packaging polymers did not affect cakes springiness factor significantly, but increasing storage time led to reduction in this property.

Resilience of packed cakes which is determined through the ratio between the areas of compression stage and decompression stage of the first cycle of texture profile analysis and is a criterion of recoverability of cakes (Fabra, Lopez‐Rubio, & Lagaron, 2015; Guadarrama‐Lezama, Carrillo‐Navas, Pérez‐Alonso, Vernon‐Carter, & Alvarez‐Ramirez, 2016). Here again, polyethylene and GNE packed cakes were more resilient than GE and GN, respectively (Table 3).

### 3.8 Sensory characterization of cakes

In order to assess organoleptic characterization of cake samples packed in gelatin, GE, GN, GNE, and polyethylene films, appearance, taste, odor, color, texture (hardness or softness), and overall acceptability of cakes were tested after 7 days of storage at 25°C (Table 2).

Results declared that cakes packed in net gelatin films had the least acceptability of sensory properties. Since net gelatin films were not a good barrier against water vapor, after 3 days of storage the cake stored in it became dried and stiff which was not chewable. On the other hand, the cakes packed in polyethylene, GE and GNE films did not possess significant difference in organoleptic characterization. By the way, polyethylene cake samples had better texture and acceptability than other cakes. It can be found from the results shown in Table 4 that the sensory properties of cakes packed in GN films were less acceptable than cakes in GE, GNE, and polyethylene films. The reason may be less capability of GN films in prevention of moisture loss and acceleration of retrogradation process of cakes in this kind of packaging. According to the findings of Jafarzadeh, Rhim, Alias, Ariffin, and Mahmud (2018), the important factors that influence food quality are moisture content, light, and oxygen exposure and these factors are impacted by food packaging. The results of their research showed that semolina films containing N‐ZnO could maintain cheese quality up to 25 days of storage at 4°C in comparison to synthetic packaging.

**Table 4:** Organoleptic properties of sponge cakes packed in gelatin, GE, GN, GNE, and polyethylene films at 25°C

Packaging film	Sensory properties					
	Appearance	Color	Odor	Taste	Texture	Overall acceptability
Gelatin	3.98^a^	8.56^a^	2.43^a^	0^a^	0^a^	3.56^a^
GE	8.56^c^	9.12^a^	7.32^c^	8.65^c^	7.73^c^	8.44^c^
GN	7.33^b^	9.34^a^	6.16^b^	6.53^b^	5.35^b^	6.33^b^
GNE	9.32^c^	9.18^a^	7.49^c^	8.43^c^	8.19^c^	8.78^c^
Polyethylene	9.06^c^	9.60^a^	8.95^c^	9.00^c^	8.50^c^	9.35^c^

Note

The results are mean of scores based on 9‐point hedonic scale (1 = dislike extremely to 9 = like extremely). The same superscripts in each column show no significant difference between values.

## 4 CONCLUSION

Substituting synthetic laminated films with laminated degradable packaging like nanocomposite gelatin/ emulsion gelatin films can achieve many advantages for industry and environment. As can be understood from the results, addition of nanoparticles to gelatin films has improved barrier properties of the films, but it was not sufficient. Accordingly, in order to lessen permeability of gases especially water vapor through packaging, applying gelatin emulsion film as the second layer could be appropriate. In this case, the results of moisture content, peroxide value, acidity, texture analysis, and fungal growth admitted that two layers nanocomposite emulsion films could be a substitute for polyethylene films.

## CONFLICT OF INTEREST

The authors declare that they do not have any conflict of interest.

## ETHICAL APPROVAL

Ethical Review: This study does not involve any human or animal testing.

Informed Consent: Written informed consent was obtained from all study participants.
